# Multifunctional role of dextran sulfate sodium for *in vivo* modeling of intestinal diseases

**DOI:** 10.1186/1471-2172-13-41

**Published:** 2012-08-01

**Authors:** William A Rose, Kaori Sakamoto, Cynthia A Leifer

**Affiliations:** 1 Department of Microbiology and Immunology, College of Veterinary Medicine, Cornell University, Ithaca, NY 14853, USA; 2 Department of Pathology, College of Veterinary Medicine, University of Georgia, Athens, GA, 30602, USA

**Keywords:** Dextran sulfate sodium, Inflammatory bowel disease, Intestinal repair, Toll-like receptor 9

## Abstract

**Background:**

Inflammatory bowel diseases (IBDs) are chronic, relapsing disorders that affect the gastrointestinal tract of millions of people and continue to increase in incidence each year. While several factors have been associated with development of IBDs, the exact etiology is unknown. Research using animal models of IBDs is beginning to provide insights into how the different factors contribute to disease development. Oral administration of dextran sulfate sodium (DSS) to mice induces a reproducible experimental colitis that models several intestinal lesions associated with IBDs. The murine DSS colitis model can also be adapted to quantify intestinal repair following injury. Understanding the mechanistic basis behind intestinal repair is critical to development of new therapeutics for IBDs because of their chronic relapsing nature.

**Results:**

The murine DSS colitis model was adapted to provide a system enabling the quantification of severe intestinal injury with impaired wound healing or mild intestinal injury with rapid restoration of mucosal integrity, by altering DSS concentrations and including a recovery phase. We showed that through a novel format for presentation of the clinical disease data, the temporal progression of intestinal lesions can be quantified on an individual mouse basis. Additionally, parameters for quantification of DSS-induced alterations in epithelial cell populations are included to provide insights into mechanisms underlying the development of these lesions. For example, the use of the two different model systems showed that toll-like receptor 9, a nucleic acid-sensing pattern recognition receptor, is important for protection only following mild intestinal damage and suggests that this model is superior for identifying proteins necessary for intestinal repair.

**Conclusions:**

We showed that using a murine DSS-induced experimental colitis model system, and presenting data in a longitudinal manner on a per mouse basis, enhanced the usefulness of this model, and provided novel insights into the role of an innate immune receptor in intestinal repair. By elucidating the mechanistic basis of intestinal injury and repair, we can begin to understand the etiology of IBDs, enabling development of novel therapeutics or prophylactics.

## Background

Inflammatory bowel diseases (IBDs) are defined as chronic, relapsing, inflammatory disorders that affect the colon and other parts of the gastrointestinal tract [1,2]. The two main types of IBDs are Crohn’s disease, which commonly causes transmural ulceration of the small intestine and colon, and ulcerative colitis, which causes non-transmural ulceration of the colon [1,2]. The annual incidence of ulcerative colitis ranges from 24.3 per 100,000 person-years in developed countries to 6.3 per 100,000 person-years in developing countries, while Crohn’s disease ranges from 20.2 to 5.0 with increasing prevalence of both IBDs observed over time [3]. IBDs develop following dysregulation of the delicate homeostatic balance maintained between the immune system of the intestine and the resident commensal bacteria [2,4-6]. The exact etiology of IBDs is unknown, but has been proposed to occur through a combination of factors, including genetic predisposition, altered expression of pattern recognition receptors, alteration in commensal microflora, and environmental stressors [2,4-6]. Prevention or treatment of IBDs is of critical importance due to the significantly increased risk of colorectal cancer, greatly diminished quality of life caused by disease symptoms, and the medical cost associated with life-long disease management [1,7,8]. Current therapeutic strategies for management of IBDs involve pharmacological interventions, including anti-inflammatory drugs, immunosuppressives, antibiotics, and biologic agents, with surgical approaches employed in critical or non-responsive patients [1,9,10]. Despite several options for IBD treatment, not all patients are responsive to therapy, and the lack of drug specificity results in several side effects complicating their use [1,5,10,11].

Current IBD treatment options lack specificity, targeting inflammation non-specifically, and lack the ability to stimulate epithelial restitution, which is important for induction of remission [1,5,10,11]. Therefore, new types of therapies are needed that can prevent disease or relapse. Several promising options are currently under evaluation in clinical trials and in different animal models of experimental colitis [1,9,12,13]. IBD-like symptoms can be chemically-induced in several animal models, but the mouse model offers several advantages including multiple methods for inducing experimental colitis, a wide array of biochemical and immunological reagents, a well-defined immune system, and the availability of numerous signaling molecule-deficient mice to evaluate treatment specificity [11,14-16]. Dextran sulfate sodium (DSS)-induced experimental colitis in mice provides a model of acute intestinal injury that mimics several intestinal lesions consistent with IBDs [14,17]. The murine DSS colitis model provides several advantages over other chemically-induced models of ulcerative colitis, including oral delivery *via* drinking water and induction of consistent colitis levels with a defined disease onset [14,16]. This model allows for investigation of environmental impacts, such as specific bacterial infections (*e.g. Campylobacter jejuni*), or exposure to probiotics [18,19]. However, most of the research on experimental colitis focuses on understanding the etiology of IBD development by studying the acute phase of intestinal injury and evaluation of methods to prevent the development of IBDs [12,20-23]. Because of the their chronic, relapsing nature and the millions of people currently affected by IBDs [1-3], there also is a need for *in vivo* models to evaluate therapeutic options that can aid in intestinal repair following IBD-induced injury.

To address the need for different models of intestinal injury and repair, we present methods to enhance the traditional DSS experimental colitis model and modifications to the model system that permit quantification of mild intestinal injury and subsequent repair. Previous studies have used severe intestinal damage models where wild-type mice are unable to repair damage during a recovery period [24-26], delivered different doses of DSS to wild-type and mutant mice in an attempt to achieve similar damage during the acute phase [27], or used intervention strategies during a recovery phase, but did not provide clinical data for the level of damage induced during the acute phase of DSS treatment [28]. One study showed that mice deficient in the negative regulator of WNT signaling had increased basal colonic epithelial cell proliferation and corresponding crypt length, and when treated with low dose DSS recovered faster than wild-type mice [29]. This suggests that using a carefully titrated dose of DSS will induce low-level damage that can be repaired rapidly, allowing comparisons between wild-type and gene-deficient mice.

We show that administration of carefully titrated concentrations of DSS to induce acute damage, and monitoring clinical and histological parameters longitudinally on a per mouse basis presented a valid system for modeling restitution of mucosal integrity. Although severity of DSS-induced colitis varies with genotype and intestinal flora, we chose to focus on C57BL/6 mice due to the availability of genetic mutants relevant to immunity on this background [19,30-34]. We show methods for presentation of clinical parameter data that are more robust than traditional disease activity indices, and describe the inclusion of several intestinal epithelial cell parameters that enhance the clarity of the data and provide insights into the potential mechanisms involved in development of intestinal lesions. Using toll-like receptor 9 (TLR9)-deficient mice, we show that by using mild intestinal damage and recovery, we could identify a novel role for TLR9 in intestinal repair, a role that was not appreciated when severe intestinal damage was induced. This approach would permit identification of genes and pathways that promote normal intestinal restitution and might be new targets for therapeutic intervention in IBD.

## Results

### Clinical parameters of the two intestinal injury models

**Figure 1:**
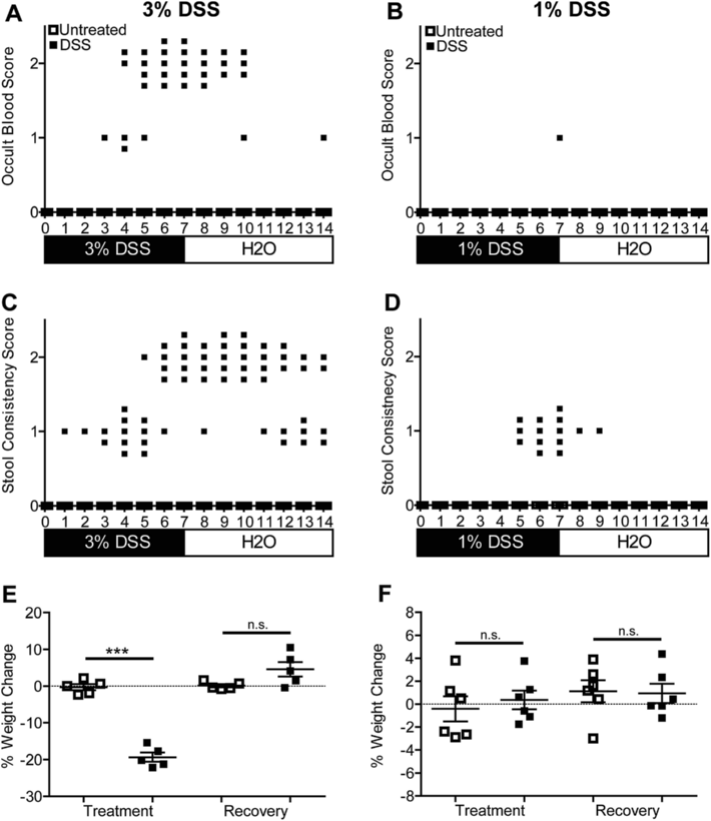
**Clinical parameter data presented for individual wild-type mice on a daily time scale.** Wild-type mice were treated with 3% **(A, C, E)** or 1% **(B, D, F)** DSS (n = 5–6 mice/group) in their drinking water for seven days, then supplied with regular drinking water for seven days (closed symbols). Control mice received normal drinking water throughout (open symbols on the X axis) Note that all 1% DSS mice scored 0 and overlap with the control mice on the X axis. Mice were scored for the presence of occult blood **(A, B)** and stool consistency **(C, D)** on a daily basis during the fourteen day experiment as described in the materials and methods. **(E, F)** Mice were weighed on a daily basis to calculate the percent change in weight for the treatment (days 0–7) and recovery (days 7–14) periods. One of two experiments (5–6 mice per condition) with similar results shown. *** *p* < 0.001; Student’s *t*-test; n.s., not significant.

Traditional data presentation methods for clinical parameters recorded during DSS-induced colitis involve a disease activity index score or an average clinical score, representing a summation of one or all clinical scores for all mice for the total experimental time [18,21-23,35]. While this value provides easily comparable numbers for identifying potential differences among groups (Table 1), this method fails to detect daily fluctuations in individual mouse parameters. By evaluating, and representing multiple parameters on a daily basis, we gain the ability to perform statistical analysis of ordinal data to identify longitudinal differences based on individual clinical parameters. Figure 1A shows daily occult blood scores for five wild-type mice treated with 3% DSS in their drinking water for seven days, followed by seven days of regular drinking water, and five wild-type (untreated) mice provided regular drinking water for fourteen days. The untreated wild-type mice did not show any signs of occult blood on any day during the course of the experiment (Figure 1A, open symbols on X axis). However, for the 3% DSS-treated wild-type mice, one mouse was Hemoccult® positive (score of 1) on day three, and by day six, all wild-type mice had visible blood in their stool (score of 2). Several wild-type mice continued to show blood in their stool even three days after cessation of DSS treatments, with one mouse testing Hemoccult® positive on day fourteen. Typical disease activity index scores shown for day 7 and day 14 (Table 1) would miss the observation that there is not an immediate resolution of occult blood following cessation of treatment. The Mann–Whitney test for ordinal data demonstrated that differences in occult blood scores between wild-type untreated and 3% DSS treated mice were statistically significant overall (*p* < 0.01), and more importantly, on a daily basis (*p* < 0.05; Days 4–10). When we compared day 7 (end of acute phase) and day 14 (recovery phase), 3% DSS-treated mice significantly improved (*p* < 0.01).

**Table 1:** Effect of DSS concentrations in wild-type mice

**Treatment**^**a**^	**N**	**Disease activity acore**	**Histologic score**
Untreated wild-type	6	0.2 ± 0.2	0.0 ± 0.0
1% DSS wild-type day 7	6	2.3 ± 0.6^b^	1.8 ± 0.4^b^
1% DSS wild-type day 14	6	0.2 ± 0.2	0.3 ± 0.2
3% DSS wild-type day 7	5	11.6 ± 0.2^b^	4.4 ± 0.5^b^
3% DSS wild-type day 14	5	2.8 ± 1.0^b^	3.8 ± 0.4^b^

^a^Mice were treated with 1 or 3% DSS in their drinking water for seven days then administered regular drinking water for an additional seven days. Clinical and histological parameters were recorded on days 7 and 14 as described in the materials and methods. Results are the mean ± standard error of the means.

^b^Significantly different from untreated wild-type mice (*p* < 0.05; Mann–Whitney test).

Two additional clinical measurements of disease activity are stool consistency and weight loss. As early as 1 day after the start of 3% DSS treatment, one mouse tested positive for loose stool (Figure 1C). By day four, all mice showed signs of loose stool, which persisted, in four out of five mice, for up to seven days after the removal of DSS. Overall (*p* < 0.001; Mann–Whitney test) and daily (*p* < 0.05; Days 4–14; Mann–Whitney test) comparisons showed significantly higher stool consistency scores for the 3% DSS treated compared to untreated wild-type mice. However, treated mice on day 14 were not improved compared to day 7 (*p* > 0.05). Wild-type mice administered 3% DSS lost significantly (*p* < 0.001; Student’s *t*-test) more weight than the untreated wild-type mice during the seven days of treatment, and showed a non-significant (*p* = 0.06; Student’s *t*-test) increase in weight during the recovery period (Figure 1E). Collectively, our data presentation method allows for quantitative and qualitative interpretation of the data to identify trends among different experimental groups. This presentation method reveals that a 3% DSS treatment model results in severe disease that is not fully repaired by seven days post-cessation of treatment.

Because IBDs are characterized by recurrent episodes of intestinal injury and repair [2,4], a model that allows for evaluation of mild intestinal injury and subsequent repair is of critical importance. Based on our data showing significant intestinal injury in mice even seven days after removal of 3% DSS (Figures 1A, C) and previous studies correlating DSS concentration with intestinal injury severity [36,37], we evaluated the clinical impact of 1% DSS in our established mouse model. Interestingly, only one of the six mice administered 1% DSS tested Hemoccult® positive on the last day of treatment (Figure 1B). No significant (*p* > 0.05; Mann–Whitney test) difference in occult blood severity was observed compared to untreated wild-type mice, while severity was significantly (*p* < 0.01; Mann–Whitney test) reduced compared to 3% DSS-treated wild-type mice (Figures 1A, B). A similar significant (*p* < 0.001; Mann–Whitney test) reduction in stool consistency scores was observed for the 1% compared to 3% DSS-treated mice (Figures 1C, D). Stool consistency in the 1% DSS-treated mice did not develop until day five, never increased past a score of 1 (loose stool), and with the exception of one mouse, scores returned to pre-treatment levels after removal of DSS (Figure 1D). Quantification of changes in weight during the treatment or recovery periods showed no significant (*p* > 0.05; Student’s *t*-test) differences between 1% DSS-treated and untreated wild-type mice (Figure 1F). Using traditional methods of disease activity indices, this 1% DSS model would not typically be characterized as disease. However, as we show below, damage is detected on a histologic level, and the significantly milder intestinal injury allows for return to baseline three days after cessation of treatment for clinical parameters, and slightly longer for histologic parameters. Therefore, the 1% DSS model is powerful method for evaluating the repair phase of IBDs.

### Histopathologic parameters of the two intestinal injury models

**Figure 2:**
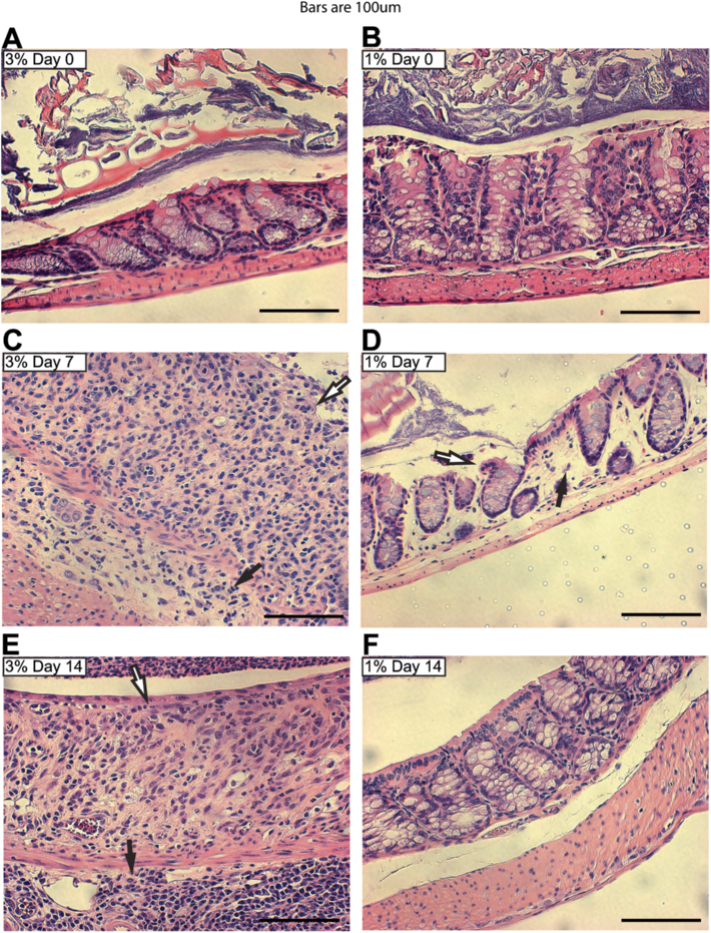
**Differences in histologic parameters between the two intestinal injury models post cessation of DSS treatments.** Wild-type mice were treated with 3% **(A, C, E)** or 1% **(B, D, F)** DSS as described in Figure 1. Colons were collected from mice on days 0 **(A, B)**, 7 **(C, D)**, and 14 **(E, F)**. Histopathologic changes in individual crypts (20x dry objective, scale bars = 100 μm) are shown in representative H&E-stained sections. Loss of crypt architecture associated with epithelial damage (white arrows) and leukocyte infiltration (black arrows) is observed following DSS treatment.

Histopathology and quantification of morphologic and immunologic changes in the colon post-DSS treatment provides another, more sensitive, measure of intestinal injury and repair [20-22,38]. Colons from 3% or 1% DSS-treated wild-type mice were collected on days zero, seven, and fourteen, then paraffin-embedded and stained with Hematoxylin and Eosin (H&E) to visualize intestinal integrity (Figure 2). Untreated wild-type mice (day zero) showed a distinctive, non-disrupted, repeating crypt architecture, with small numbers of leukocytes present in the lamina propria (Figure 2A, B). After seven days of 3% DSS treatment, however, wild-type mice showed mucosal erosions (white arrow) with effacement of the mucosa by lymphocytes, neutrophils, and macrophages, which also infiltrate and expand the submucosa (black arrow; Figure 2C). These changes indicate severe disease, consistent with severe observed clinical disease (Figure 1). Despite the mild clinical intestinal injury observed in the 1% DSS-treated wild-type mice, histopathology showed significant loss of crypts (white arrow, Figure 2D), and expansion of the lamina propria by edema fluid after seven days (black arrow, Figure 2D). However, by seven days after 1% DSS removal (day fourteen), there was complete restitution of normal colonic architecture (Figure 2F). In contrast, and consistent with maintained clinical indices of disease seven days post-cessation of 3% DSS treatment, we observed continued mucosal effacement, with early attempts at repair through neovascularization and surface re-epithelialization (white arrow, Figure 2E), as well as increase submucosal infiltration by lymphocytes and plasma cells (black arrow, Figure 2E). Together, the histopathologic observations indicate that the 1% DSS-treated wild-type mice experienced mild intestinal injury that they were able to repair within seven days post cessation of treatment, while 3% DSS treatment induce extensive intestinal injury that showed markedly delayed repair during a seven day recovery period. However, histopathology is often subjective and not presented in a quantitative, or statistically amenable form.

**Figure 3:**
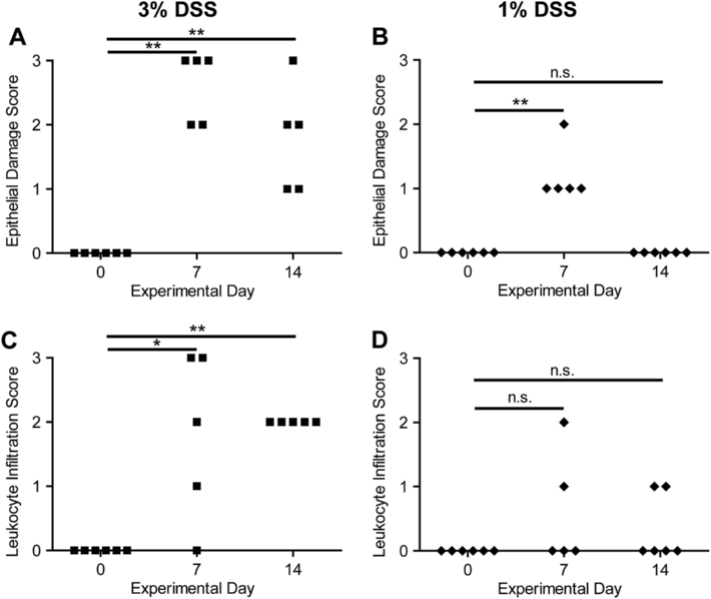
**Quantification of histologic parameters collected from individual wild-type mice before, during, and after DSS treatment.** H&E-stained colon sections were collected as described in Figure 2 and scored in a blinded manner by a board-certified veterinary pathologist. **(A, B)** Epithelial damage was scored as: 0) no damage; 1) discrete focal lymphoepithelial lesions; 2) mucosal erosion; or 3) mucosal erosion with submucosal damage. **(C, D)** Leukocyte infiltration was scored as: 0) rare inflammatory cells in the lamina propria; 1) inflammatory cells throughout most of the lamina propria; 2) inflammatory cells extending into the submucosa; or 3) transmural extension of inflammatory cells. * *p* < 0.05, ** *p* < 0.01; Mann Whitney test; n.s., not significant.

Quantitative scoring of histopathology by a board-certified veterinary pathologist using a previously described scoring system [39], showed no epithelial damage or leukocyte infiltration in the wild-type mice at day zero (Figure 3). Seven days of treatment with 3 or 1% DSS resulted in significantly (*p* < 0.01; Mann–Whitney test) increased epithelial damage scores compared to day zero (Figures 3A, B). At day 14, seven days after changing to normal drinking water, the epithelial damage scores for 3% DSS-treated wild-type mice were still significantly (*p* < 0.01; Mann–Whitney test) increased (Figure 3A). In contrast, epithelial damage scores returned to pre-treatment levels in mice that were treated with 1% DSS (Figure 3B). A significant increase in leukocyte infiltration scores for 3% DSS-treated mice was observed on days seven (*p* < 0.05; Mann–Whitney test) and fourteen (*p* < 0.01; Mann–Whitney test) (Figure 3C). This indicated that the inflammatory response to tissue damage was still occurring at this time point. In contrast, mice treated with 1% DSS did not show significantly (*p* > 0.05; Mann–Whitney test) higher leukocyte infiltration scores on either day seven or fourteen (Figure 3D). Scoring analysis supported the histologic observations that treatment of wild-type mice with 3% DSS induced a severe intestinal injury that failed to repair within one week of cessation of DSS treatment, while 1% DSS treatment induced milder injury that was readily repaired. This milder treatment model, along with the statistical analysis of quantitative histopathologic data, provides a system to evaluate the role of various parameters in intestinal repair.

### Intestinal epithelial cell parameters of the two intestinal injury models

**Figure 4:**
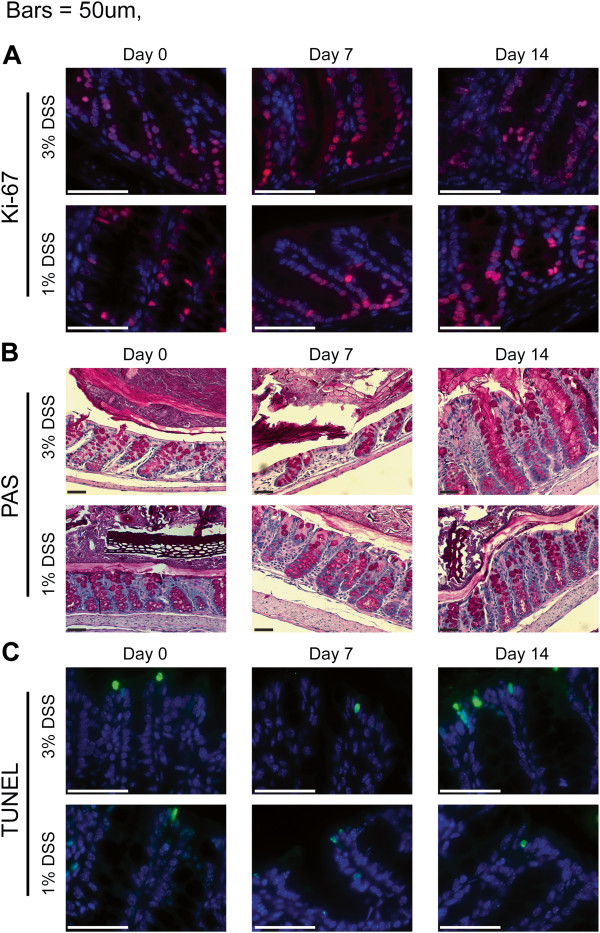
**Representative quantitative histology images. **Images from colonic sections of 3% or 1% DSS-treated, wild-type mice collected on days 0, 7, and 14. **(A)** Sections were stained for Ki-67 (red) and DAPI (blue) and imaged with a 63x oil objective. **(B)** Sections were stained with PAS for goblet cells and imaged with a 20x dry objective. **(C)** Sections were TUNEL-stained (green) for apoptotic cells, counterstained for DAPI (blue), and imaged with a 63x oil objective. All scale bars = 50 μm.

**Figure 5:**
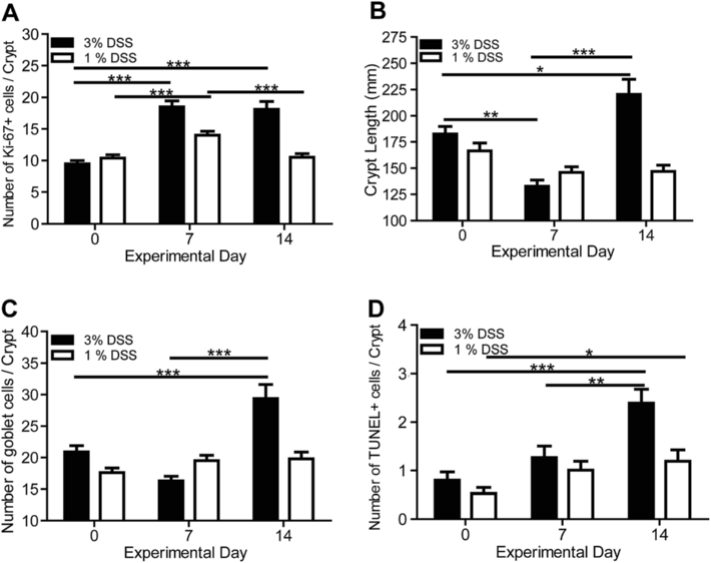
**DSS treatment induced quantitative changes in colonic epithelial cell populations.** Colonic sections were collected from 3 or 1% DSS-treated, wild-type mice on days 0, 7, and 14. 50 crypts from three mice at each time point for each DSS treatment (150 crypts at each treatment time point) were analyzed for: **(A)** number of Ki-67+ cells per colonic crypt, **(B)** colonic crypt length, **(C)** goblet cells per colonic crypt, and **(D)** number of TUNEL + cells per colonic crypt. Bars represent mean ± standard error of the means. Values were not significant unless indicated: * *p* < 0.05, ** *p* < 0.01, *** *p* < 0.001; ANOVA.

Intestinal epithelial cells play a critical role in maintenance of intestinal homeostasis following injury [20,40,41]. Therefore, we propose that in addition to quantifying clinical and histologic parameters in the DSS colitis model, changes in intestinal epithelial cell parameters should also be quantified to provide potential insights into the mechanisms underlying responses to intestinal injury and repair. Based on several published works showing differences in intestinal epithelial proliferation following induction of intestinal injury [20,42,43], we quantified the number of Ki-67+ cells present in colon crypts (Figures 4A and 5A). Wild-type mice showed a significant (*p* < 0.001; ANOVA) increase in Ki-67+ cells after seven days of treatment with 3% or 1% DSS (Figure 5A). Seven days post-DSS treatment, wild-type mice administered 3% DSS still exhibited significantly (*p* < 0.001; ANOVA) elevated numbers of Ki-67+ cells while 1% DSS-treated wild-type mice returned to pre-treatment numbers. One potential explanation for the increased numbers of Ki-67+ cells following induction of intestinal injury is a compensatory increase in colon crypt length following initiation of repair; therefore we quantified crypt lengths (Figures 4B and 5B). As expected, a significant (*p* < 0.01; ANOVA) reduction in crypt length was observed for mice treated with 3% DSS for seven days; however, a slight, but non-significant (*p* > 0.05; ANOVA), decrease was observed for 1% DSS-treated mice (Figure 5B). After recovery (day fourteen), the 3% DSS-treated mice showed a significant (*p* < 0.05; ANOVA) increase in crypt length (Figure 5B). This correlated with significantly increased numbers of goblet cells present in the crypts (Figures 4B and 5C). While TUNEL + cells per crypt were increased at day 14 for both 3% (*p* < 0.001; ANOVA), and 1% (*p* < 0.05; ANOVA), DSS-treated mice, no dramatic increases in apoptosis were observed overall (Figures 4C and 5D). Collectively, the quantitative intestinal epithelial data shows that severe intestinal injury (3% DSS) produces significant and prolonged changes at the level of individual cells, while mild injury (1% DSS) produces only minor changes with a rapid return to a pre-injury state.

### A role for TLR9 in intestinal repair is only observed in a mild intestinal injury model

**Figure 6:**
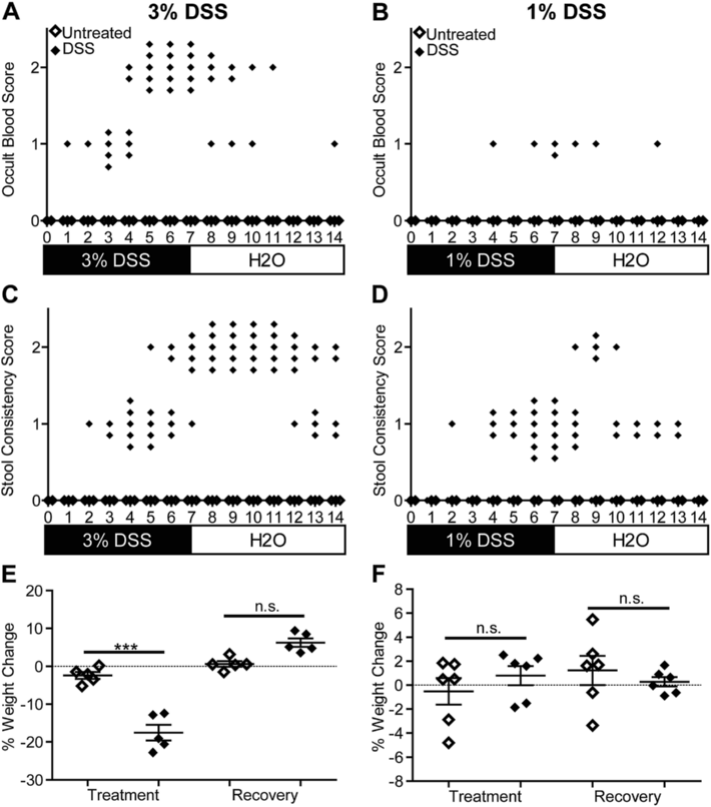
**TLR9 is important for protection against mild, but not severe intestinal injury.** TLR9-deficient mice were treated with 3% **(A, C, E)** or 1% **(B, D, F)** DSS in their drinking water for seven days, then supplied with regular drinking water for seven days. Wild type mice from the same experiment are depicted in Figure 1 for comparison. Mice were scored for the presence of occult blood **(A, B)** and stool consistency **(C, D)** on a daily basis during the fourteen day experiment as described in the materials and methods. (E, F) Mice were weighed on a daily basis to calculate the percent change in weight for the treatment (days 0–7) and recovery (days 7–14) periods. One of two experiments (5–6 mice per condition) with similar results shown. *** *p* < 0.001; Student’s *t*-test; n.s., not significant.

Mice lacking MyD88, a critical innate immune signaling molecule [44], exhibited altered severity to DSS-induced colitis indicating an important role for innate immune signaling in intestinal injury [20]. However, the role of TLRs, most of which signal through MyD88 [44], in intestinal repair following injury needs to be evaluated further. By using the two different DSS models of intestinal injury, the mechanistic role of TLRs, for instance TLR9, can be quantified on a clinical, histologic, and epithelial cell level. TLR9-deficient mice treated with 3% DSS for seven days then provided regular drinking water for seven days showed significantly (*p* < 0.001; Mann–Whitney test) increased occult blood scores compared to untreated TLR9-deficient mice (Figure 6A, control wild-type mice from the same experiment are depicted in Figure 1A). Occult blood scores of 3% DSS-treated TLR9-deficient mice were not significantly different from 3% DSS-treated wild-type mice (*p* > 0.05; Mann–Whitney test). Furthermore, the development and progression of occult blood was very similar between wild-type and TLR9-deficient mice (Figures 1A and 6A). Similar results were obtained for stool consistency scores. TLR9-deficient mice treated with 3% DSS showed significant increased stool consistency scores (*p* < 0.001; Mann–Whitney test), but these were not different from 3% DSS-treated wild-type mice (Figures 1C and 6C). As with the wild-type mice (Figure 1E), treatment of TLR9-deficient mice with 3% DSS produced a significant (*p* < 0.001; Student’s *t*-test) reduction in total body weight during the treatment period, with a significant (*p* < 0.01; Student’s *t*-test) increase observed during the recovery period (Figure 6E). Using this model, we would conclude that TLR9 plays no role in intestinal damage or repair induced by DSS.

To determine whether a role for TLR9 in intestinal damage and repair was masked by such severe intestinal damage induced by the 3% DSS treatment regimen, we employed the 1% DSS mild intestinal injury model. Interestingly, the TLR9-decifient mice treated with 1% DSS not only showed significantly (*p* < 0.01; Mann–Whitney test) increased occult blood scores compared to untreated TLR9-deficient mice (Figure 6B), they also scored significantly (*p* < 0.05; Mann–Whitney test) higher than similarly treated wild-type mice (control wild-type mice from the same experiment depicted in Figure 1B). By using daily analysis, we observed that one 1% DSS-treated TLR9-deficient mouse tested Hemoccult® positive as early as day four after beginning DSS treatment, and remained positive as late as five days after removal of DSS (Figure 6B). These observations would be missed using standard disease indices as described above (Figure 1, Table 1). The stool consistency scores for the 1% DSS-treated TLR9-deficient mice were also significantly (*p* < 0.001; Mann–Whitney test) increased over both untreated TLR9-deficient mice, and 1% DSS-treated wild-type mice (*p* < 0.05; Mann–Whitney test) (Figures 1D and 6D). Initial onset of loose stool was noted on day two for one 1% DSS-treated mouse, with several mice showing very loose stool (score = 2) after DSS cessation and continued clinical outcomes until day thirteen (Figure 6D). Again, these subtle differences would not be noted in a traditional disease index score evaluation. The 1% DSS-treated TLR9-deficient mice did not show any significant (*p* > 0.05; Student’s *t*-test) change in weight compared to untreated TLR9-deficient or 1% DSS-treated wild-type mice during the treatment or recovery periods (Figures 1F and 6F). The increased severity, earlier onset, and delayed disappearance of clinical outcomes in the TLR9-deficient mice, indicate that TLR9 signaling plays an important role in protection against mild intestinal injury and in repair.

## Discussion

While there are several therapeutic options for inducing remission of IBDs [1,9,10], some patients are refractory to treatment or exhibit adverse side effects [1,5,10,11], therefore a critical need exists for new intervention strategies. Current research focuses on elucidating the etiology of IBDs [12,20-23], but due to the chronic, relapsing nature of IBDs and the millions of people currently affected by IBDs [1-3], research aimed at improving our understanding of intestinal repair processes after injury is also necessary. To address this issue, we showed a method for generating two different reproducible models of intestinal lesion development by administering selected concentrations of DSS in mice. Additionally, we provided new methods to analyze clinical parameter data in addition to inclusion of epithelial cell parameter data. We also show the importance of the two different model systems for evaluating the contribution of pattern recognition receptors towards preventing intestinal injury and in stimulating repair. The two different model systems provide methods that will enhance our understanding of the mechanistic basis of IBD development and intestinal repair following injury.

Oral delivery of DSS in mice results in the development of experimental colitis that models several clinical signs and lesions associated with IBDs [14,17]. Based on the proposed epithelial cell toxicity of DSS [45], we showed that increasing concentrations of DSS in the regular drinking water of mice resulted in increasing severity of intestinal injury (Figures 1, 2, 3). The reproducible nature of DSS-induced experimental colitis allowed for optimization of DSS concentrations to provide two model systems consisting of: 1) a model of the severe intestinal lesions and impaired would healing associated with IBD lesions, and 2) a model of mild intestinal injury with subsequent repair. The severe intestinal injury or acute model is important for identifying factors that are involved in the development of IBDs [2,4-6]. The mild intestinal injury and repair model is a complementary approach that may reveal novel functions of proteins that are obscured when severe intestinal damage is induced [1,2]. Utilization of the two different model systems will provide a better understanding of the temporal development of intestinal lesions and resulting clinical outcomes that are hallmarks of IBDs.

Representation of clinical parameters obtained during DSS-induced experimental colitis in mice vary greatly from a single disease activity index score to an average score for each disease parameter based on daily or total experimental time [18,21-23]. Issues with this type of data presentation include obscuring potential differences in disease outcomes, lack of temporal context for progression of the disease, potential outliers within the population, and summation of discrete ordinal data mixed with continuous data hypothesis testing. Separate presentation of each measured clinical parameter allows for more complete understanding of the extent of disease outcome as it relates to different types of intestinal lesions (Figure 1). Coupled with temporal presentation of the individual clinical parameters, the progression of intestinal lesions can be quantified and provide insights into the initiating events that are critical to understanding the development of IBDs. Because most clinical parameters are scored on a graded integer scale [18,21-23], summation of the data for the group of mice can result in a non-integer score that creates confusion as to the exact clinical outcome. Presentation of the individual data points for each mouse avoids the issue and hypothesis testing using non-parametric methods allows for statistical quantification of the data (Figure 1). The new methods we have provided for presentation of clinical data parameters offers a novel format for effectively and quickly conveying information that is relevant to understanding how intestinal lesions develop and progress during intestinal injury and repair.

The intestinal epithelium is composed of different types of epithelial cells that are important for normal host biology and providing protection against development of intestinal diseases [20,40,46]. Therefore, understanding how these cell populations are potentially altered following intestinal injury and repair is of critical importance. We show that after seven days of 3% DSS treatment in wild-type mice, colon crypts were significantly ablated and most of the remaining epithelial cells were actively proliferating to compensate for loss of intestinal barrier integrity (Figures 4 and 5). After removal of DSS, the proliferative cells continued to divide and crypt lengths increased significantly by day 14 to provide more absorptive surface area and the cells needed to repair the lesions [40]. An increase in goblet cell numbers also was observed and correlates with the known role of goblet cell mucins and intestinal trefoil factors in providing protection against the development of experimental colitis and initiation of intestinal repair processes in mice [47-49]. Additionally, an increase in the number of apoptotic cells was observed as expected based on the proposed intestinal epithelial cell toxicity of DSS [45]. Together, the quantified intestinal epithelial cell parameters show that the specific epithelial cell populations are dramatically altered following intestinal injury and induction of repair.

Pattern recognition receptors play a critical role in maintaining intestinal homeostasis, and alteration of receptor expression has been implicated as one of the factors involved in the development of IBDs [50]. To quantify the potential involvement of a nucleic acid sensing pattern recognition receptor, we evaluated TLR9-deficient mice in both intestinal injury models (Figure 6). The results from TLR9-deficient mice treated with 3% DSS indicate that TLR9 does not play a role in protection against severe intestinal injury or a potential contribution is obfuscated by the severity of the intestinal damage. Interestingly, evaluation of TLR9-deficient mice in the mild intestinal injury model (1% DSS) showed that TLR9 signaling is important for protection against injury and is involved in repair. Other TLRs and their adaptor molecules were shown to provide protection against intestinal injury through different mechanisms of action and MyD88, specifically, was shown to contribute to intestinal repair [12,20,42,51]. Notch signaling is required for the differentiation of intestinal epithelial progenitor cells, and NF-kB activation contributes to the initiation of intestinal repair. Both signaling pathways are activated following TLR stimulation [40,52]. Therefore, TLRs, such as TLR9, likely play a critical role in normal host biology and provide protection against chronic intestinal disease through the induction of intestinal repair.

## Conclusions

We show that mild DSS-induced experimental colitis in mice permits evaluation of various effectors, including TLR9, in protection from intestinal damage, and for induction of intestinal restitution. We provide a new method for presenting clinical parameter data that highlights individual mouse disease outcomes over the course of the experiment, and that permits statistical analysis of differences on a daily or total experimental time basis. We demonstrate methods for quantification of changes in the intestinal epithelial cell populations, following intestinal injury and repair, to determine the etiologies of observed intestinal lesions. Knowledge of the mechanistic basis resulting in intestinal lesion development associated with IBDs will provide key insights into the development of novel therapeutic strategies. This same information will also provide insights into the normal host intestinal functions in the context of mild inflammation and loss of epithelial barrier function.

## Methods

### Mice

C57BL/6 (WT) and TLR9 receptor-deficient mice were 8–12 weeks old and weighed an average of 20 grams. The mice were bred in the Cornell University Transgenic Mouse Core Facility and housed under specific pathogen-free conditions. This animal study was approved by the Cornell University Institutional Animal Care and Use Committee (IACUC).

### Colitis induction

Wild type and TLR9-deficient mice, in the same experiment, were administered DSS (36,000-50,000 Da; MP Biomedical, Solon, OH) *ad libitum* in drinking water at 1% or 3% weight per volume. Fresh 1% or 3% DSS was supplied on days 3 and 5 for treated mice. Control mice received regular drinking water for all fourteen days of the experiment. For the purposes of data analysis, wild type mice are depicted in Figure 1 and TLR9-deficient mice are depicted in Figure 6; however, the mice were from the same experiment.

### Clinical scoring

Weight, stool consistency, and presence of occult blood (Hemoccult® SENSA®, Beckman Coulter, Brea, CA) were scored daily as previously described [38]. Briefly, scores for stool consistency were: 1) soft, but still formed; 2) very soft stool; or 3) diarrhea. Scores for occult blood were: 1) a positive Hemoccult® test; 2) visible blood in the stool; or 3) rectal bleeding. Total % weight change during treatment was calculated as: % Weight Change = [(weight at day 7 – weight at day 0)/weight at day 0] × 100 and recovery as: [(day 14 – day 7)/day7] × 100.

### Colon collection and histopathology scoring

On days 0, 7, and 14, mice were euthanized and whole colons were excised from the anus to the cecum. The colons were coiled starting at the cecum, then fixed in 10% formalin (Azer Scientific, Morgantown, PA) for 24 hours. The fixed colons were paraffin-embedded and sectioned (5 μm), then H&E or periodic acid-Schiff (PAS) stained at the Cornell University Animal Health Diagnostic Center. Blinded scoring of H&E-stained sections was performed by a board-certified veterinary pathologist as previously described [39].

### Quantitative histology of colon sections

Sections from paraffin-embedded colons were deparaffinized with three, two minute washes in xylene (Sigma, St. Louis, MO), then rehydrated with two minute washes in a graded ethanol series (Sigma) of 100, 90, and 75%. For heat-induced epitope retrieval, slides were microwaved at 800 watts for 20 minutes in a 10 mM sodium citrate pH 6.0 solution with 0.05% Tween-20 (Sigma). The *In Situ* Cell Death Detection kit, Fluorescein (Roche, Indianapolis, IN) was used to identify apoptotic cells in colonic sections. KI-67 positive cells were labeled using a Mouse on Mouse kit (Vector, Burlingame, CA) with a monoclonal Ki-67 (Clone MM1; Leica, Buffalo Grove, IL) primary antibody and a streptavidin-conjugated Alexa Fluor 555 (Life Technologies, Grand Island, NY) detection antibody. Slides were counterstained with Prolong Gold Anti-fade Reagent with DAPI (Life Technologies), then imaged using an Axio Imager M1 microscope (Zeiss, Thornwood, NY) and an Axiocam MRm (Zeiss). To quantify crypt length and goblet cell numbers, H&E (crypt length) or PAS (goblet cells)-stained colonic sections were imaged using an Axio Imager M1 microscope and an Axiocam HRc (Zeiss). Crypt length was quantified by measuring from the basolateral surface of the basal crypt cells to the apical cell surface at the top of intact crypts using the length function in the AxioVision Rel. 4.8 software (Zeiss). A total of 50 measurements were collected for each quantitative outcome.

### Statistical analysis

Statistical analyses were performed using Prism software package v5 (Graphpad, La Jolla, CA) as described in the figure legends.

## Abbreviations

DSS: Dextran sulfate sodium; H&E: Hematoxylin and eosin; IBD: Inflammatory bowel disease; PAS: Periodic acid-Schiff; TLR9: Toll-like receptor 9.

## Competing interests

The authors declare that they have no competing interests.

## Authors’ contributions

WR and CL designed the experiments, WR performed the experiments, KS performed pathology analysis and edited the manuscript, and WR and CL interpreted the data and wrote the manuscript. All authors read and approved the final manuscript.
